# Supplementary material for: The interactome of intact mitochondria by cross-linking mass spectrometry provides evidence for coexisting respiratory supercomplexes
Source: Mol Cell Proteomics. 2017 Dec 8;17(2):216–32. doi: 10.1074/mcp.RA117.000470 (PMC5795388; doi:10.1074/mcp.RA117.000470)
Supplement: Supplemental Data [file supp_RA117.000470_133922_0_supp_23978_fzffwf.zip › spectra_annotation/mito_DR_spectra_annotation/188-1-1-1-2-1.pdf]

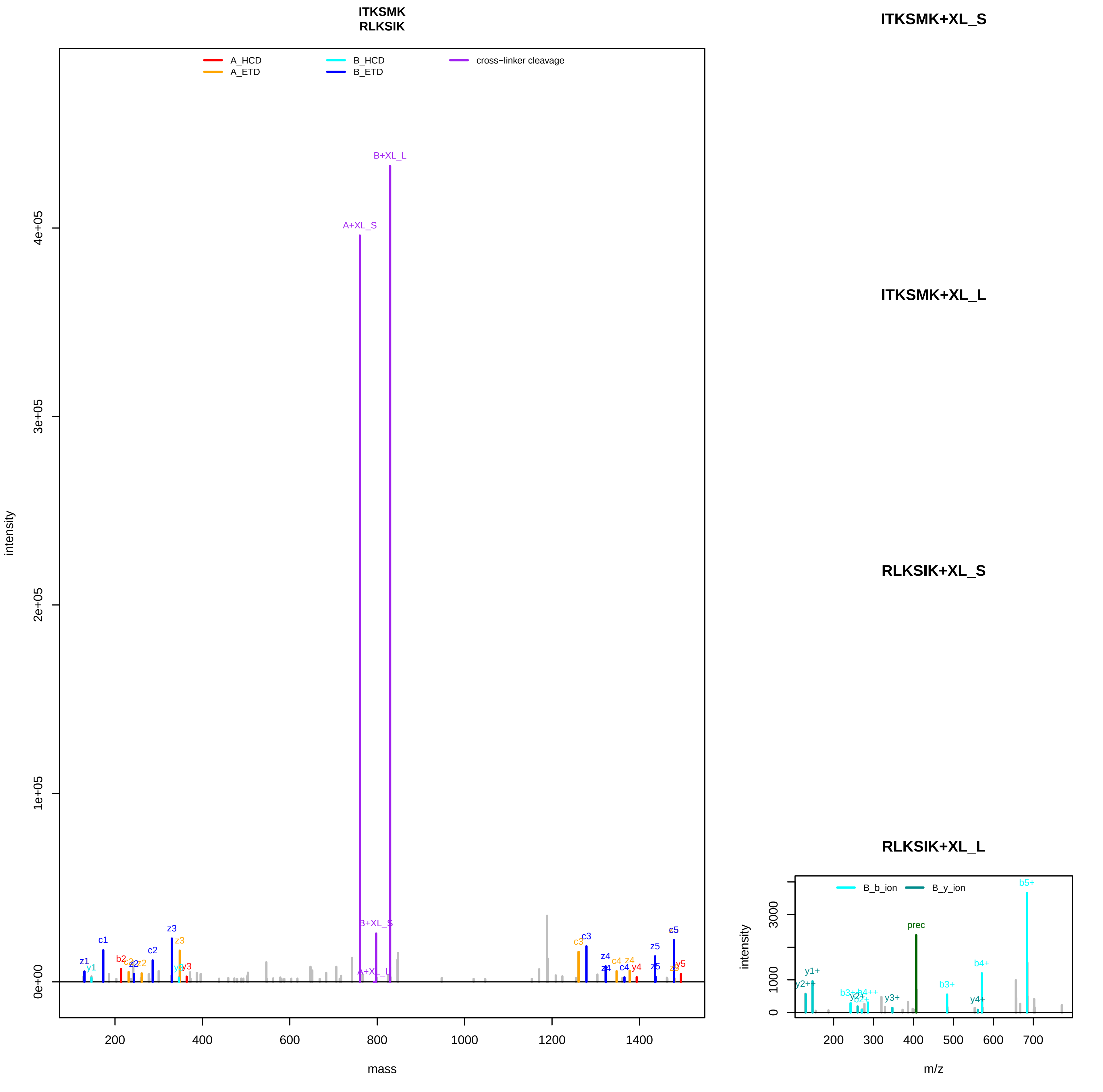
ITKSMK
RLKSIK
A_HCD
A_ETD
B_HCD
B_ETD
cross−linker cleavage
B+XL_L
A+XL_S
B+XL_S
A+XL_L
intensity
mass
0e+00
1e+05
2e+05
3e+05
4e+05
200
400
600
800
1000
1200
1400
ITKSMK+XL_S
ITKSMK+XL_L
RLKSIK+XL_S
RLKSIK+XL_L
B_b_ion
B_y_ion
b5+
prec
b4+
b3+
y1+
y2++
y3+
y4+
m/z
0
1000
3000
200
300
400
500
600
700
